# Rest in Cardiac Affections

**Published:** 1894-04

**Authors:** 


### REST IN CARDIAC AFFECTIONS.

Dr. T. Lauder Brunton (*The Practitioner*) believes that as a remedial measure, rest frequently requires to be absolute; as a preventive one it may be relative. The amount enjoined must be carefully proportioned to each case, as in advanced mitral disease, when the power of the heart is failing, absolute rest gives satisfactory results, in that the circulation recovers its balance, the arteries become filled and the veins emptied, the dropsical effusion and venous engorgement of the organs disappear, and the patient recovers a fair amount of health. In cases of mitral disease incompetence may come about from :

1. Enlargement of the auriculo-ventricular orifice.
2. Thickening of the valves, or
3. Incoördinated action of the musculi papillares.

In the first case it may be hard to say if this be the sole cause of the regurgitation, without any obvious disease of the valves, as some disturbance of the relationship between the musculi papillares may tend to aid the regurgitation. In such hearts in growing boys and in chlorotic girls, comparative rest may be useful, and sometimes absolute rest may be almost essential. In some cases the former may be all that is wanted as a prophylactic measure. In chlorotic girls gentle exercise is advisable, but it must be carefully graduated, as exhaustion is likely to do harm. Massage may be useful, as it gives the patient exercise without putting any strain upon the heart. With a fatty heart gentle exercise may be advisable, as it may be more likely to bring about a healthy condition of the heart than absolute rest. When in mitral disease cardiac tonics, even pushed to their utmost limit, fail to give relief, then absolute rest becomes of great importance. Massage is of great usefulness in clearing out the body-waste, quickening the flow of blood through the muscles, and relieving the edema, and the patient gets the advantage of the exercise without overdoing his heart. It also allows the treatment to be carried out more easily than it would otherwise be, for it removes the feeling of weariness and irritability, faintness and unrest of the patient.—*Medical Review*, February 10, 1894.

---

It is estimated that about 400 candidates will apply for the diploma of the Board of Regents in the State of New York, during the coming year. If the enterprising medical student alluded to in the last number, succeeds in getting the fee reduced to $5.00, it will be impossible for the Board to do this work, unless the State steps in and pays this expense. At the time the law was passed, the State said it must be a paying board. Now the lawyers want the medical examiners to work for nothing, *for the honor of the thing*, as one of the members of the committee who heard the delegates, from the State Medical Society, suggested. We would like to know about how much labor lawyers do for the honor of the thing, without any money. No doubt it is true that the medical profession is bound to do a great deal without any reward other than comes from the consciousness of doing its duty, but we think senators and assemblymen are a little hard on them when they propose that we should select seven examiners to travel all over the State, prepare a series of carefully arranged questions on seven subjects, examine and decide on four hundred answers, for the honor of the work.—*Post-Graduate*, March, 1894.

